# Assessment of cardiovascular physiology using magnetic resonance myocardial stress testing reveals impaired contractile reserve in patients with cirrhotic cardiomyopathy

**DOI:** 10.1186/1532-429X-17-S1-Q67

**Published:** 2015-02-03

**Authors:** Francisco Sampaio, Pablo Lamata, Nuno Bettencourt, Sophie-Charlotte Alt, Nuno D Ferreira, Johannes T Kowallick, Joana Pimenta, Shelby Kutty, Jose Fraga, Paulo Bettencourt, Vasco Gama, Andreas Schuster

**Affiliations:** 1 Cardiology Department, Centro Hospitalar Gaia/Espinho, VN Gaia, Portugal; 2 University of Porto Medical School, Porto, Portugal; 3 Biomedical Engineering, King's College London, London, UK; 4 Department of Pediatric Cardiology and Intensive Care Medicine, Georg-August University, Göttingen, Germany; 5 Institute for Diagnostic and Interventional Radiology, Georg-August University, Göttingen, Germany; 6 German Centre for Cardiovascular Research, Göttingen, Germany; 7 Children's Hospital and Medical Center, University of Nebraska Medical Center, Omaha, NE; 8 Gatsroenterology Department, Centro Hospitalar Gaia/Espinho, VN Gaia, Portugal; 9 Division of Imaging Sciences and Biomedical Engineering, The Rayne Institute, Kings College London, London, UK; 10 Department of Cardiology and Pneumology, Georg-August University, Göttingen, Germany

## Background

Liver cirrhosis has been shown to affect cardiac performance. However cardiac dysfunction may only be revealed under stress conditions. The value of non-invasive stress tests in diagnosing cirrhotic cardiomyopathy is unclear since their ability to detect abnormalities has been inconsistent using different imaging modalities. We sought to investigate the response to pharmacological stimulation with dobutamine in patients with cirrhosis using cardiovascular magnetic resonance.

## Methods

Thirty-six patients and eight controls were studied. Conventional volumetric and myocardial deformation parameter analysis using feature tracking at rest and during low to intermediate dose dobutamine stress were performed.

## Results

Whilst volumetry based parameters were similar between patients and controls at rest, patients had a smaller increase in cardiac output during stress (2.2 l/min vs. 3.8 l/min), p=0.015). Chronotropic response was not different in the two groups (24 bpm vs 25 bpm, p=0.44). Ejection fraction increase was impaired in patients during 10 μg/Kg/min dobutamine as compared to controls (6.9% vs. 16.5%, p=0.007), but not with 20 μg/Kg/min (12.1% vs. 17.6%, p=0.12). This was paralleled by an impaired improvement in circumferential strain with low dose (median percentual increase of 14.4% vs. 30.9%, p=0.03), but not with intermediate dose dobutamine (median percentual increase of 29.4% vs. 33.9%, p=0.54). There was an impaired longitudinal strain increase in patients as compared to controls during low (median percentual increase of 6.6% vs 28.6%, p<0.001) and intermediate dose dobutamine (median percentual increase of 2.6%vs, 12.6% p=0.016). Radial strain response to dobutamine was similar in patients and controls (median percentual increase of 7.7% (-2.4-15.2) vs 13.6% (5.7-26), p=0.11 with10 μg/Kg/min of dobutamine and 4.3% (0.6-8.1) vs 3.1% (-0.2-9.3), p=0.82 with 20 μg/Kg/min of dobutamine).

## Conclusions

Cirrhotic cardiomyopathy is characterized by an impaired cardiac pharmacological response that can be detected with magnetic resonance myocardial stress testing. Deformation analysis parameters may be more sensitive in identifying abnormalities in inotropic response to stress than conventional methods.

## Funding

NA.

